# Caspian Red Deer Population Dynamics Under Changing Climate Conditions in Central Alborz: Two Decades of Conservation En Route to Restoration

**DOI:** 10.1002/ece3.71799

**Published:** 2025-07-20

**Authors:** Farid Salmanpour, Zahra Shakoori, Mahan Salmanpour, Mehdi Kia, Rahman Eshaghi, Abolfazl Rahbarizadeh, Faraham Ahmadzadeh

**Affiliations:** ^1^ Department of Biodiversity and Ecosystem Management Environmental Sciences Research Institute, Shahid Beheshti University Tehran Iran; ^2^ Department of Natural Resources – Environment Islamic Azad University, Science and Research Branch Tehran Iran; ^3^ Department of Environment Mazandaran Provincial Office Mazandaran Iran

**Keywords:** Central Alborz, Golestanak core zone, habitat restoration, humidity, snowfall, temperature, wildlife conservation

## Abstract

Climate change and habitat loss are among the most significant threats to global biodiversity, profoundly altering species distributions, reproductive success, and the number of individuals. This study focuses on the Caspian red deer ( *Cervus elaphus maral* ) within the Golestank core zone of the Central Alborz Protected Area (CAPA), investigating how climatic variables and conservation interventions have influenced their numbers over the past two decades. By integrating comprehensive field surveys, demographic assessments, and weather data from regional weather stations, we identified a consistent annual growth rate of 2.2%. This positive trend is attributed to enhanced calf survival and a stable male‐to‐female ratio, reflecting the population's resilience under conservation management. Warmer winter temperatures were found to positively influence population numbers, whereas increased spring snowfall exerted a detrimental effect. These findings highlight the importance of targeted habitat conservation efforts to buffer the impacts of climate change and support long‐term population sustainability.

## Graphical abstract

Climate change and habitat loss are among the most significant threats to global biodiversity, profoundly altering species distributions, reproductive success, and the number of individuals. This study focuses on the Caspian red deer ( *Cervus elaphus maral* ) within the Golestank core zone of the Central Alborz Protected Area (CAPA), investigating how climatic variables and conservation interventions have influenced their numbers over the past two decades. By integrating comprehensive field surveys, demographic assessments, and climate data from regional weather stations, we identified a consistent annual growth rate of 2.2%.

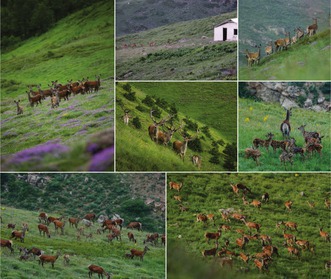

## 1 Introduction

Climate change is increasingly recognized as a major driver of ecosystem transformations, exerting profound effects on species distribution, reproductive success, and the number of individuals (Iler et al. 2021; Santini et al. 2021; Franklin 2023; Howard et al. 2024; Lawlor et al. 2024). These impacts are particularly pronounced for species already under pressure from habitat loss and fragmentation (Deb et al. 2020; Román‐Palacios and Wiens 2020; Pigot et al. 2023; Rubenstein et al. 2023; Wudu et al. 2023; Divieso et al. 2024). The synergistic effects of anthropogenic climate change and habitat degradation are driving unprecedented biodiversity declines globally, disrupting ecological processes and altering both intraspecific and interspecific interactions (Brambilla et al. 2020; Åkesson et al. 2021; Fisher et al. 2021; Ferretti and Fattorini 2021; Nielsen et al. 2021; Chen et al. 2023). Such disruptions are often expressed through changes in behavior, foraging strategies, and migration patterns, which can significantly reduce species resilience and push many closer to vulnerability or extinction (Beale et al. 2023; Findlay‐Robinson et al. 2023; Moura et al. 2023; Khera et al. 2023; Verzuh et al. 2023). This highlights the urgent need for research to unravel the complex interplay between climate change and other anthropogenic pressures, in order to inform effective conservation strategies.

The intensification of anthropogenic disturbances, including poaching, habitat fragmentation, and climate change, has significantly contributed to mammal extinctions during the Anthropocene, with larger species being disproportionately affected (Santini et al. 2016; Pacifici et al. 2017; Ripple et al. 2017; Crooks et al. 2017; Bowyer et al. 2019; Wan et al. 2019; Romero‐Muñoz et al. 2020). Large herbivores are particularly vulnerable due to their reliance on climate‐sensitive vegetation for sustenance and their high value as a food source for humans (Dobbins et al. 2020; Lovari et al. 2020; Veldhuis et al. 2020; Bachmann et al. 2022). The increasing frequency and intensity of extreme weather events, such as abrupt changes in temperature, humidity, and precipitation, have further disrupted the reproductive success, age structure, sex ratios, and survival of calves of large herbivores (Kerby and Post 2013; Froy et al. 2019; Renaud et al. 2019; Pérez‐Barbería et al. 2020; Paoli et al. 2020; Tyler et al. 2020; Millán et al. 2021).

In response to these challenges, many terrestrial species are migrating long distances to locate suitable habitats, with northern latitudes and higher elevations often serving as critical refuges (Årevall et al. 2018; Rivrud et al. 2019; Butt et al. 2021; Williams et al. 2022; Ramalho et al. 2023). High‐altitude grasslands, which may exhibit enhanced fertility under warming conditions, offer vital resources for these migrating populations (Su et al. 2018; Ye et al. 2018; Bedoya‐Durán et al. 2023; Ramalho et al. 2023). However, human activities in the Anthropocene, such as infrastructure expansion and land‐use changes, present formidable obstacles to these migratory movements (Salmanpour, Shakoori, et al. 2025). These barriers exacerbate the vulnerability of large terrestrial mammals to the impacts of climate change, posing significant threats to their survival (Tucker et al. 2018; Wan et al. 2019; Doherty et al. 2021; Davison et al. 2021; Paniw et al. 2022; Abrahms et al. 2023; Pacifici et al. 2020; Haight et al. 2023; Anderwald et al. 2024).

Among cervids, large species such as red deer ( *Cervus elaphus* ) exhibit heightened sensitivity to climate change due to their specific habitat requirements. Environmental variables, including altitude, temperature, humidity, and precipitation, play a critical role in shaping their distribution, behavior, and ecological strategies (Mysterud et al. 2008; Stopher et al. 2014; Dawe and Boutin 2016; Kennedy‐Slaney et al. 2018; Weiskopf et al. 2019; Jiang et al. 2020; Niedziałkowska et al. 2021; Salmanpour et al. 2023). Red deer require large home ranges to facilitate long‐distance movements, particularly altitudinal migrations, which are essential for accessing optimal birthing habitats with suitable thermal conditions and sufficient food resources (Luccarini et al. 2006; Rivrud et al. 2010; Van Beest et al. 2011; Smolko et al. 2018; Bojarska et al. 2020; Salmanpour et al. 2022; Jarnemo et al. 2023).

The Caspian red deer ( *Cervus elaphus maral* ), an endemic subspecies in Iran, has suffered a staggering population decline of over 80% within the past four decades. This decline is driven by a combination of factors, including illegal hunting, habitat loss, competition with domestic livestock, and the growing impacts of climate change. Unlike European red deer populations, which are classified as Least Concern, the Caspian red deer's precarious situation has resulted in its designation as Endangered. This stark contrast underscores the critical need for intensified conservation efforts to safeguard this vulnerable subspecies (Kiabi et al. 2004; Lovari et al. 2018; Soofi et al. 2018; Yusefi et al. 2019; Shokri et al. 2021).

Established in 1994, the Golestanak Ranger Station has played a pivotal role in wildlife conservation by enforcing a livestock grazing ban, restricting unauthorized human access, and implementing robust anti‐poaching measures. These efforts have created a high‐altitude sanctuary crucial for local wildlife. Each year, the majority of the red deer population in CAPA migrates to the Golestanak core range in late spring, where they complete critical life stages such as calving in the spring and mating in late summer. Following these vital periods, they return to the lower‐elevation winter pastures in the northern part of the forest in early autumn, maintaining seasonal movement patterns that are essential for their survival and reproductive success (Faghihi et al. 2022; Mazandaran Bureau of the Department of Environment 2023; Salmanpour et al. 2023; Nezami et al. 2024). As climate change reshapes environmental conditions, Caspian red deer are increasingly expanding beyond protected areas and historical ranges. This shift raises concerns about intensified poaching pressure, particularly during the mating season, when males become more vulnerable due to their conspicuous behavior. Both sexes generally move toward more favorable habitats to meet their ecological needs; however, during certain periods, females may ascend to higher elevations, possibly to reduce predation risk and to find and secure suitable calving sites (Ziaei 2008; Rivrud et al. 2019; Jiang et al. 2020; Salmanpour et al. 2022).

Iran's strategic location in a climate change hotspot amplifies the combined threats of escalating poaching pressures and habitat loss, posing a multifaceted challenge to endangered species, particularly the Caspian red deer (Yousefi et al. 2019; Shokri et al. 2021; Yusefi et al. 2019, 2022). The lack of long‐term, cohesive studies on the population dynamics of red deer in Iran has created considerable uncertainty regarding the future viability of this species, highlighting the need for comprehensive research to inform effective conservation strategies.

Longitudinal population data from both protected areas and wild habitats provide critical insights into the trajectory and rate of climate change impacts, as well as the effectiveness of conservation efforts. This study utilizes over two decades of comprehensive population data collected from the Golestanak core zone in northern Iran, supplemented by daily meteorological records from nearby weather stations. We examined changes in the population size of red deer over recent years, assessing the NDVI, climatic, and temporal factors influencing population fluctuations. Additionally, we explored trends in key population dynamics parameters, including the number of males, females, and calves, as well as the male‐to‐female and calf‐to‐female ratios. Furthermore, we investigated the climatic, temporal, and spatial factors affecting the red deer population within the Central Alborz Protected Area (CAPA). By addressing these critical questions, our research seeks to fill a significant gap in ecological knowledge, contributing to the development of informed conservation strategies essential for safeguarding the Caspian red deer amidst the growing challenges posed by climate change and human‐induced pressures.

## 2 Materials and Methods

### 2.1 Study Area

The Central Alborz Protected Area (CAPA) in Mazandaran province, Iran, is a significant biodiversity hotspot, situated at the convergence of the eastern and western populations of the Alborz Mountains and the Hyrcanian forests, a UNESCO World Heritage site (Naderi et al. 2012; UNESCO World Heritage Centre 2019; Noroozi et al. 2019, 2020).

**FIGURE 1:**
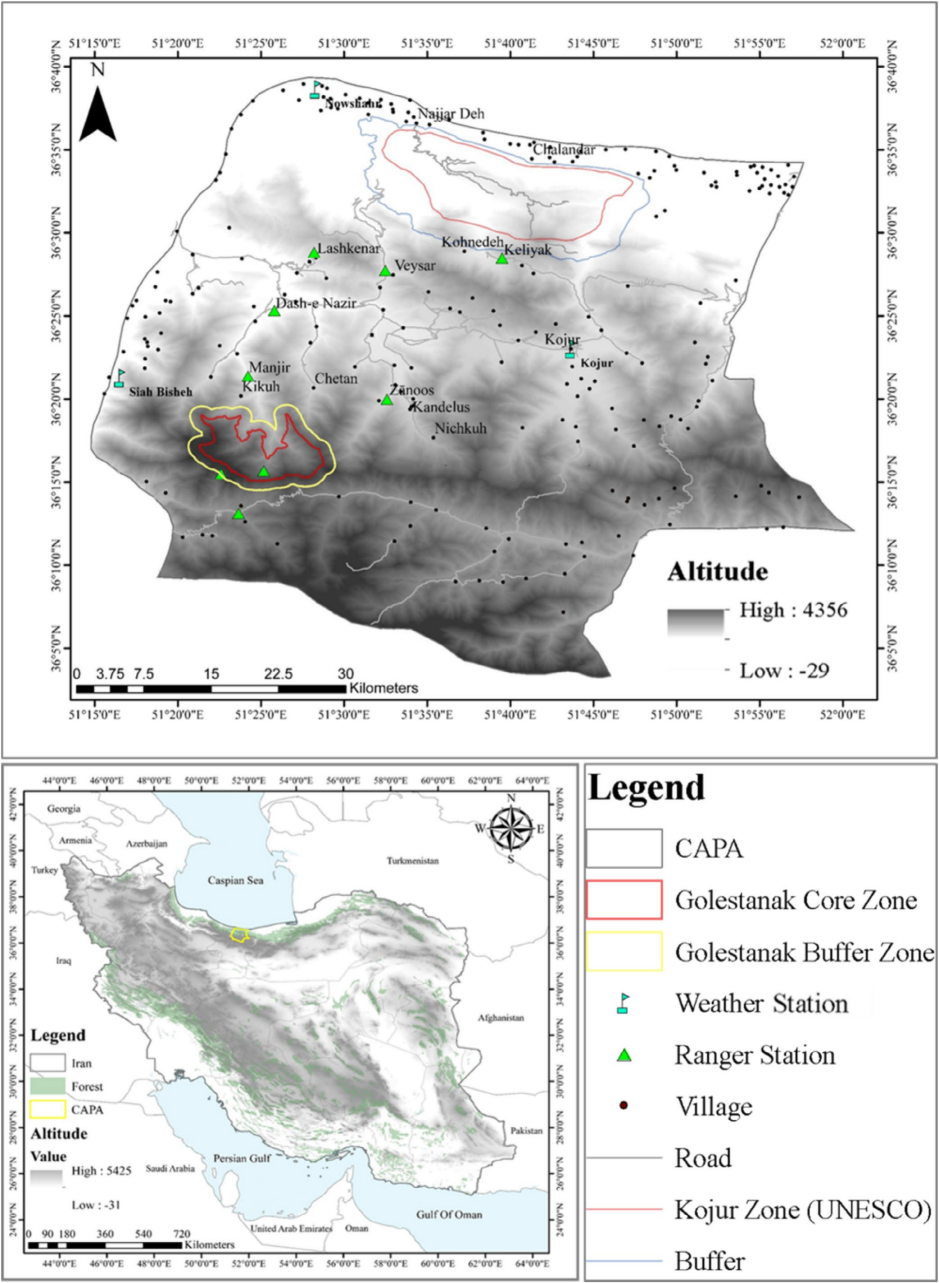
Map showing Iran (lower panel) with the location of the Central Alborz Protected Area (CAPA), where this study was conducted. The top panel provides a detailed view of CAPA, highlighting the Golestanak Core Zone, villages, roads, and ranger stations (Salmanpour, Shakoori, et al. 2025).

Spanning 2950 km^2^, CAPA encompasses a diverse landscape, ranging from the northern slopes of the Alborz Mountains, exceeding 4000 m in elevation, to the low‐lying coastal plains bordering the Caspian Sea at just 16 m below sea level (Figure 1). Vegetation types vary accordingly, from dense Hyrcanian forests at lower elevations to the more sparse and Alborz‐specific vegetation found at higher altitudes (Darvishsefat 2008). CAPA is a crucial habitat for numerous endangered species, including the Caspian red deer, Persian leopard (*Panthera pardus tulliana*), brown bear ( *Ursus arctos* ), and roe deer ( *Capreolus capreolus* ), and a diverse assemblage of other large mammals also native to Iran, such as the gray wolf ( *Canis lupus* ), wild goat ( *Capra aegagrus* ), and wild boar ( *Sus scrofa* ). The area is managed by 13 ranger stations, encompassing three inactive, three seasonal, three semi‐active, and four active stations. However, the proximity of over 220 villages and over 400 km of paved road creates a conservation paradox. While this close proximity suggests a degree of human‐wildlife coexistence, it also presents serious challenges, including conflicts arising from carnivore attacks on livestock, illegal hunting, livestock grazing, and habitat alteration, all of which pose significant threats to the area's biodiversity (Nezami and Farhadinia 2011; Karami et al. 2016; Soofi et al. 2018; Yusefi et al. 2019; Salmanpour et al. 2021, 2023; Ashrafzadeh et al. 2022; Ghoddousi and Khorozyan 2023).

Since 1994, livestock grazing and unauthorized human activities have been strictly prohibited within the Golestanak core zone of CAPA. Prior to this period, the area was subjected to unrestricted livestock grazing and frequent human movement, which undoubtedly exerted considerable pressure on the ecosystem. The implementation of stringent protection measures following the prohibition has been instrumental in mitigating the adverse effects of past disturbances. Over the past decades, the cessation of livestock grazing and the restriction of access to all but a limited number of authorized individuals, such as rangers, experts, and researchers, has facilitated the natural regeneration and recovery of the habitat (Mazandaran Bureau of the Department of Environment 2022).

### 2.2 Data Collection

This study examined the population dynamics of Caspian red deer within the Golestanak core area, a key highland refuge located in the Central Alborz Protected Area (CAPA), over the summer periods (15 May to 20 September) from 1995 to 2022.

Given the inherent challenges associated with monitoring endangered species characterized by low population densities and elusive behavior, particularly within remote and rugged highland environments such as those of CAPA, specific methodological measures were implemented to minimize sources of error and uncertainty. To ensure data reliability and comparability across years, field surveys were conducted following standardized protocols. These included: (i) training and calibrating methods of observers to a consistent skill level; (ii) utilizing uniform data recording equipment; (iii) maintaining fixed patrol routes and survey designs across years; and (iv) implementing long‐term annual monitoring with repeated sampling. Previous studies have demonstrated that adherence to such rigorous field protocols can substantially reduce observational errors and improve the robustness of population estimates under challenging field conditions (Krebs 1972, 1989; Regan et al. 2002; Sutherland 2006).

#### 2.2.1 Population Data

The Caspian red deer population within the Central Alborz Protected Area (CAPA) is estimated at approximately 300–350 individuals, with a significant proportion (70%–80%) migrating seasonally to the Golestanak core zone between late spring and early autumn. Within this core zone, the population is estimated at 240–260 individuals. This seasonal movement is primarily driven by the ecological importance of Golestanak as a critical breeding and calving habitat, providing optimal conditions for parturition and early‐stage offspring development (Faghihi et al. 2022; Mazandaran Bureau of the Department of Environment 2023; Salmanpour et al. 2023; Salmanpour, Shakoori, Keshtkar, et al. 2025).

Throughout the critical reproductive season, the Golestanak core area has been subject to intensive and continuous wildlife monitoring, supported by the permanent presence of rangers stationed at the Golestanak Ranger Station. From late May through early October each year, rangers maintained 24 h surveillance, while stringent conservation measures including the prohibition of domestic livestock grazing, rigorous anti‐poaching enforcement, and restrictions on human access ensured minimal disturbance and enhanced habitat security for the red deer population (Salmanpour, Shakoori, Salmanpour, et al. 2025; Salmanpour, Shakoori, Keshtkar, et al. 2025).

The high‐altitude environment of Golestanak (ranging from 2200 to 4000 m a.s.l.), combined with the availability of nutrient‐rich summer pastures, provided ideal conditions for conducting systematic and uninterrupted daily wildlife observations over the 27‐year study period. Data collection focused on key demographic parameters, including age and sex structure, with individuals visually classified into three categories: adult males, adult females, and newborn calves based on distinctive morphological characteristics, body size, and other visible markers commonly employed in ungulate field studies.

#### 2.2.2 Observation Techniques

**FIGURE 2:**
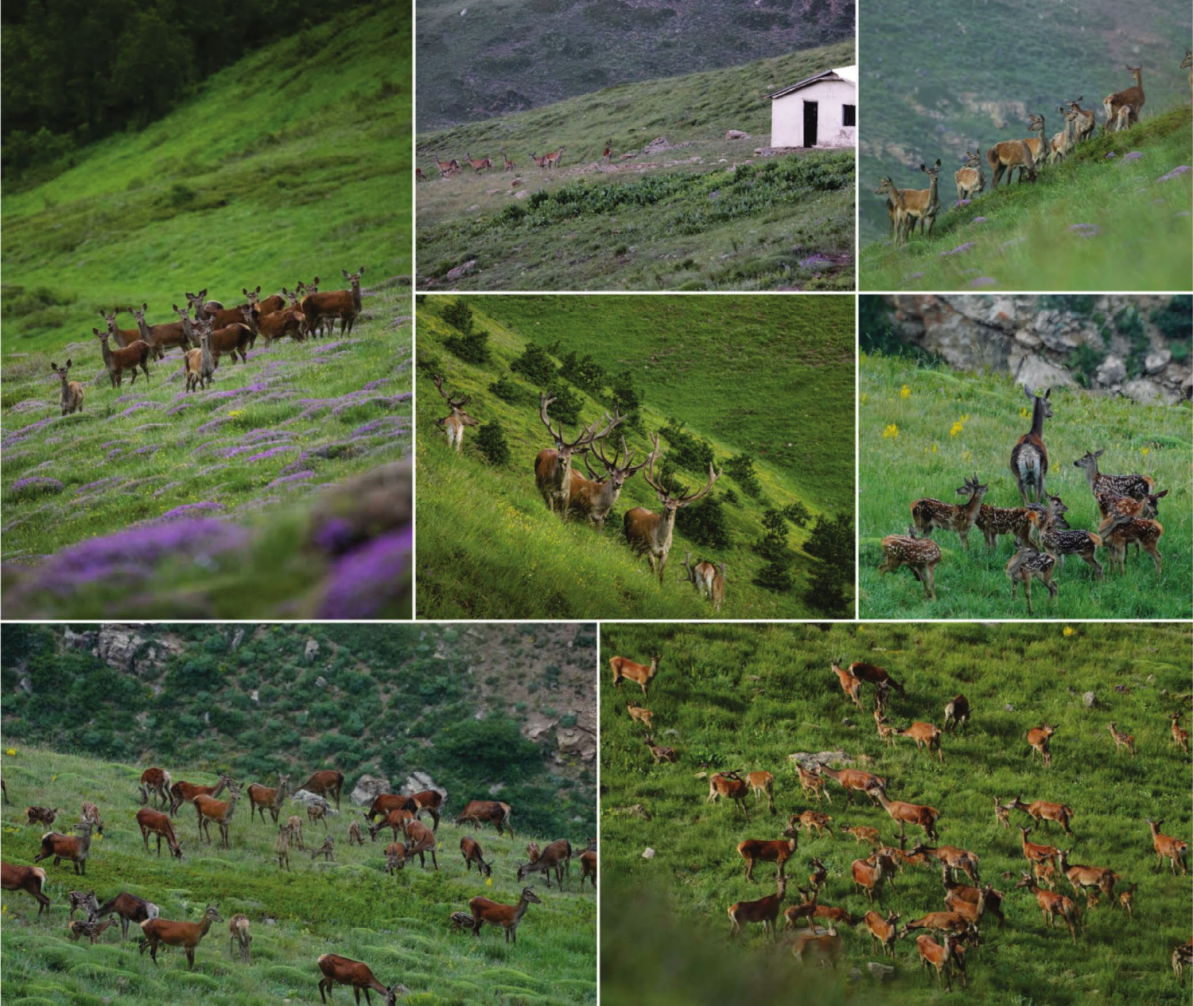
Landscape of the study area in the Golestanak core zone. The images show male and female red deer in their natural habitat, with an elevation range of 2200–4000 m above sea level. The Golestanak ranger station is also visible in one of the corners of the images, providing context for the location.

Systematic daily observations were conducted using binoculars and digital photography, in accordance with standardized wildlife monitoring protocols recommended for large mammal species (Hefley et al. 2013; Airst and Lingle 2020). The calves have reddish‐brown hair with clearly defined white spots that are distinct in size and pattern, serving as key morphological features for age identification (Ziaei 2008; Karami et al. 2016). This methodology is particularly suitable for monitoring large‐bodied species that aggregate seasonally, as supported by earlier ecological field studies (Krebs 1972, 1989). The open alpine vegetation structure of the Golestanak region, characteristic of the High Alborz landscape (Figure 2), provided favorable visibility conditions that enhanced the probability of detection during field surveys (Darvishsefat 2008; Naderi et al. 2012, 2018).

Field observations were carried out through structured daily patrols, typically conducted by two‐person teams comprising at least one senior ranger to ensure consistent observer expertise across survey efforts. Patrol routes were standardized and repeated annually, a practice shown to improve data reliability and reduce observer bias in long‐term wildlife monitoring programs (Krebs 1989; Hochachka et al. 2000; Burton 2012; Afriyie et al. 2021).

#### 2.2.3 Standardized Data Collection Protocol

To ensure consistency, accuracy, and comparability of population data across survey years, all observational records from daily patrols were systematically documented by the lead ranger responsible for each patrol session. Field observers employed standardized data recording sheets provided by the Iranian Department of Environment (DOE), specifically designed to capture essential ecological and demographic parameters.

For each observation event, detailed information was recorded, including the date, species identity, total number of individuals observed, and key demographic attributes such as sex and age class (adults and newborn calves). The variables recorded were straightforward and clearly defined, further reducing the potential for observer bias. The standardized classification of individuals facilitated consistent data categorization across observers and years. The implementation of this structured protocol aimed to minimize observational bias and enhance data quality, in line with established best practices for ecological monitoring (Krebs 1972, 1989; Sutherland 2006).

#### 2.2.4 Monitoring Effort and Data Validation

Between 1995 and 2022, a total of 3384 monitoring days were conducted in the Golestanak core area. These figures indicate the number of days each group was directly observed during standardized patrols. Long‐term monitoring, combined with repeated daily observations, is a well‐established method for reducing uncertainties in wildlife studies. Averaging counts across multiple days minimizes the effects of temporary movements and detection variability, increasing the accuracy and reliability of population estimates (Krebs 1989).

#### 2.2.5 Weather Data

Daily climatic data were collected from three synoptic weather stations: Siah Bisheh (2200 m a.s.l.), Nowshahr (−3 m a.s.l.), and Kojur (1500 m a.s.l.). The Siah Bisheh station, located closest to the Golestanak core zone, provided climate information comparable to the study region, while the other stations offered broader climatic context for CAPA. Key climatic variables analyzed included mean daily temperature (°C), relative humidity (%), daily precipitation (mm), and maximum snowfall (mm). These parameters were selected based on their documented influence on ungulate behavior and population dynamics (Olson et al. 2015; Borowik et al. 2016; Bonardi et al. 2017; Pelaez et al. 2017; Froy et al. 2019; Pérez‐Barbería et al. 2020; Millán et al. 2021). Seasonal mean weather parameters were calculated by first averaging the daily values of each parameter within each season: winter (December, January, and February), spring (March, April, and May), summer (June, July, and August), and autumn (September, October, and November) for each year.

#### 2.2.6 Normalized Difference Vegetation Index (NDVI)

Satellite remote sensing is increasingly used to supplement terrestrial monitoring, and in some regions, is the only available data source. The MODIS Normalized Difference Vegetation Index (NDVI), produced every 16 days at multiple spatial resolutions, provides consistent measurements of vegetation cover, leaf area, chlorophyll content, and canopy structure. Derived from atmospherically corrected reflectance in the red, near‐infrared, and blue bands, MODIS NDVI values are obtained at 250–500 m resolution, representing a 16‐day average per pixel. Data selection criteria include low cloud cover (< 5%) and the highest NDVI value (Didan 2021). We calculated the average NDVI for each summer (May–August) from 1996 to 2022 to analyze long‐term vegetation trends in the study area.

### 2.3 Statistical Analyses

#### 2.3.1 Linear Regression Analysis

To evaluate long‐term temporal trends in climatic variables, we applied linear regression models within a 95% confidence interval. Linear regression is widely regarded as a robust and appropriate method for detecting directional changes in ecological time‐series data, particularly for environmental variables that are expected to respond gradually and consistently over time (Weisberg 2005; Seber and Lee 2012; James et al. 2023). In all models, key climatic parameters were treated as dependent variables, with year included as a continuous independent variable to quantify potential linear trends.

#### 2.3.2 Quadratic Regression Analysis

In contrast, population dynamics in large wildlife species, such as red deer, are often influenced by complex, nonlinear processes driven by a combination of environmental and ecological factors. To account for these potential nonlinearities, we applied quadratic regression models within a 95% confidence interval. Quadratic regression offers a flexible and powerful framework for modeling ecological responses that may exhibit curvilinear patterns, thresholds, or intermediate optima, rather than simple linear trajectories (Matsui 2020). Moreover, this approach is particularly valuable for detecting and interpreting population–environment relationships in noisy and dynamic ecological systems (Wood 2010). In all models, mean red deer abundance across demographic groups was modeled as the dependent variable, with year and year‐squared terms included as continuous independent variables to capture both linear and nonlinear components of temporal population trends.

#### 2.3.3 Pearson Pairwise Correlation Analysis

To evaluate interrelationships among climatic variables, we conducted Pearson pairwise correlation analyses at the 95% confidence level, following the procedure outlined by Teng and Chen (2024). Climatic predictors were selected based on either their strong correlation with other variables or their statistical independence. This selection strategy ensured that the most representative variables were retained while minimizing multicollinearity.

#### 2.3.4 Mixed‐Effects Model

The mixed‐effects model offers a robust statistical framework that allows for the separation of fixed and random effects, making it particularly suitable for analyzing complex ecological data where variability arises from both known (fixed) and unknown (random) factors (Pinheiro and Bates 2000). In this study, we employed this model to investigate the factors influencing the population change trends of the Caspian red deer.

To assess the influence of various factors on these population trends, we utilized the Restricted Maximum Likelihood (REML) estimation method. This method enabled us to model year as a random effect, the Normalized Difference Vegetation Index (NDVI) and climate parameters as covariates, specifically evaluating their impact on changes in red deer population dynamics. The climatic variables considered included seasonal mean temperature (°C), snowfall (mm), relative humidity (%), and rainfall (mm) for winter, spring, and summer. NDVI was included as a key covariate, reflecting vegetative cover and its influence on deer populations. The response variable in our analysis was the total population size (i.e., males, females, and calves).

REML is a particularly advantageous method for ecological data analysis, as it allows for the accurate estimation of variance components in mixed‐effects models, even when data are unbalanced or where random effects play a significant role. This approach provides unbiased estimates of fixed effects, while enhancing model accuracy, making it well‐uited for ecological research (Marra and Wood 2011; Nakagawa and Schielzeth 2013; Froy et al. 2019; Pérez‐Barbería et al. 2020).

#### 2.3.5 Inverse Distance Weighting (IDW)

We conducted spatial analysis and map generation using ArcGIS 10.8 to enhance the interpretation of temperature variations across CAPA. The study area was divided into 5 × 5 km grid cells, a resolution selected to provide a balanced approach for large‐scale red deer habitat management (Georgii 1980; Salmanpour, Shakoori, Salmanpour, et al. 2025; Salmanpour, Shakoori, Keshtkar, et al. 2025). Spatial maps were developed in ArcGIS 10.8, integrating red deer distribution data from Salmanpour et al. (2022), Mazandaran Bureau of the Department of Environment (2023). Regions with a higher likelihood of red deer presence were mapped and overlaid onto climate output maps to examine potential spatial overlaps between habitat use and climatic conditions. To model annual temperature fluctuations, data from three meteorological stations were interpolated using the inverse distance weighting (IDW) method in ArcGIS 10.8, applying the following formula:

$$Z\left(x\right)=\frac{\sum_{i=1}^{n}\frac{z_{i}}{d_{i}^{p}}}{\sum_{i=1}^{n}\frac{1}{d_{i}^{p}}}$$

Where, $Z\left(x\right)$ is the interpolated value, $z_{i}$ represents the known values, ${\mathrm{d}}_{i}$ is the distance between the known and interpolated points, p is the power parameter controlling the influence of distance, and n is the number of known points, was applied to produce descriptive maps. This approach visualized spatial temperature patterns across the study area, offering valuable insights into the relationship between climatic variability and Caspian red deer habitat distribution.

#### 2.3.6 Anderson–Darling Test

The normality of the annual population data were assessed using the Anderson–Darling test, which indicated a normal distribution (*p* > 0.05).

## 3 Results

**FIGURE 3:**
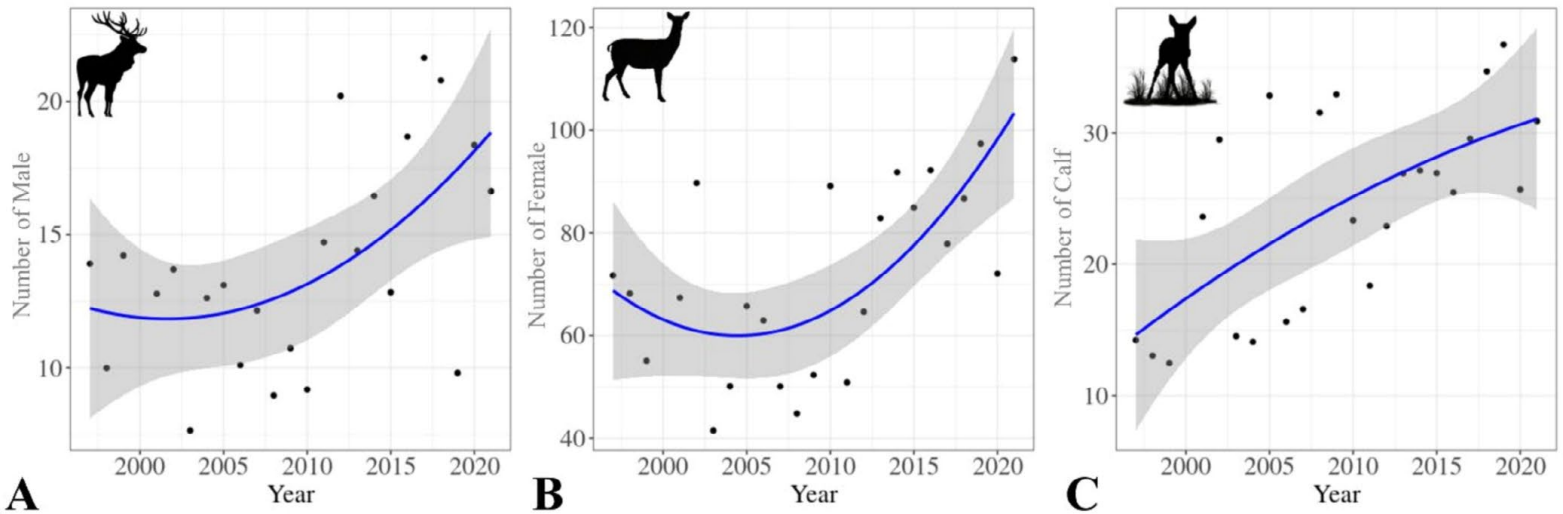
Quadratic regression trends showing population changes of (A) male deer, (B) female deer, and (C) calves.

The quadratic regression results showed significant temporal trends in the population structure of red deer. Our findings showed a significant temporal trend in the male population (Figure 3A) over the years (*p* < 0.001). The model explained 49% of the variance (*R* ^2^ = 49%), with a strong overall fit (*F* = 18.8, df = 24). The regression analysis (Figure 3B) revealed a significant relationship between year and the female population (*p* < 0.001). The model accounted for 46.7% of the variance (*R* ^2^ = 46.7%), with a solid overall fit (*F* = 13.2, df = 24). A significant temporal trend was observed in the calf population over the years (*p* = 0.003). The model explained 50.26% of the variance (*R* ^2^ = 43.17%), with an excellent overall fit (*F* = 24, df = 23) (Figure 3C).

**FIGURE 4:**
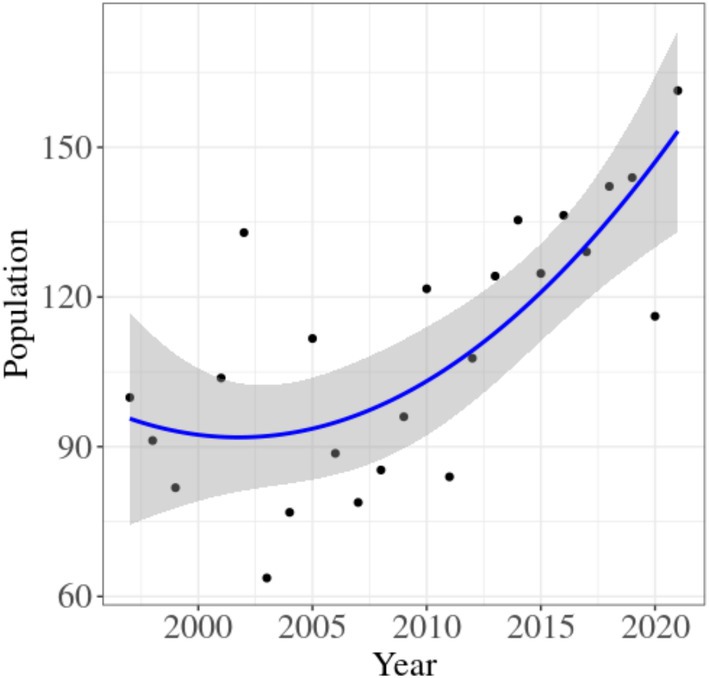
Quadratic regression of red deer population over time in the Golestanak core area, indicating an overall increasing trend.

Quadratic regression analysis revealed a significant upward trend in total population size over the study period (*p* < 0.001, *R* ^2^_adj_ = 53.43%). Males increased by an average of 2.1% annually, females by 2.2%, and calves by 3.0%. The overall population showed an annual growth rate ranging between 1.6% and 2.1%, reflecting a steady demographic expansion (Figure 4).

Between 1997 and 2000, the number of observed males ranged from 10 to 14 individuals, increasing to 16–19 individuals between 2018 and 2022. The female segment of the population also exhibited a marked rise, with counts increasing from 55 to 71 individuals in the earlier period to 87–113 individuals in the latter. Notably, the number of calves experienced the most substantial increase, rising from 12 to 23 individuals to 25–36 individuals over the same timeframe. Consequently, total population size increased from a range of 81–103 individuals during 1997–2001 to 116–162 individuals during 2018–2022, indicating consistent growth across all demographic groups over the 26‐year period. It is important to note that these figures represent the minimum population size observed within the Golestanak core zone.

### 3.1 Calf‐to‐Female Ratio

**FIGURE 5:**
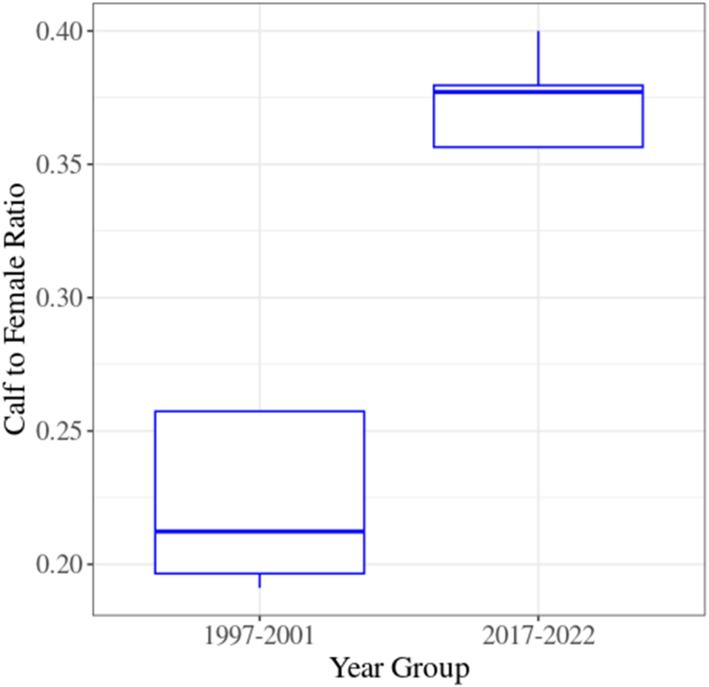
Boxplot comparing the calf‐to‐female ratio between the first and last 5 years of the study period.

The calf‐to‐female ratio was calculated to determine whether, alongside the increase in calf and female populations in recent years, the calf‐to‐female ratio has also increased, potentially indicating relative reproductive success (Figure 5). In the first five years of the study (1997–2001), an average of approximately 0.25 calves per female was recorded, increasing by about 40% to 0.35 calves per female in the final 5 years (2017–2022).

### 3.2 Male‐to‐Female Ratio

The regression analysis showed no significant temporal trend in the male‐to‐female ratio (*p* = 0.769). The model explained no variance (*R* ^2^ = 0%), with a negligible overall fit (*F* = 0.27, df = 24). This stability suggests that the male‐to‐female ratio has remained relatively consistent over the years, likely due to intrinsic population structures or balanced survival rates across sexes.

### 3.3 Pearson Correlation Analysis

Based on the results of the Pearson correlation analysis, we initially considered all seasonal climatic variables. We selected parameters that either showed strong correlations with other variables (and served as representatives) or were independent and uncorrelated with the rest. From the winter parameters, Winter‐rrr24, Winter‐tm, and Winter‐essmax were selected; from spring, Spring‐essmax and Spring‐tm; and from summer, NDVI, Summer‐um, and Summer‐rrr24. Other variables were excluded due to redundancy or lack of correlation with any variable, indicating limited contribution to the model (Table 1).

**TABLE 1:** Results of the Pearson correlation test between seasonal weather variables.

Sample 1	Sample 2	*N*	Correlation	95% CI for *ρ*	*p*
Winter‐rrr24	Winter‐tm	21	0.090	(−0.356, 0.502)	0.698
Winter‐essmax	Winter‐tm	20	0.024	(−0.423, 0.462)	0.920
Winter‐um	Winter‐tm	21	−0.404	(−0.712, 0.033)	0.069
Winter‐essmax	Winter‐rrr24	21	0.322	(−0.128, 0.662)	0.155
Winter‐um	Winter‐rrr24	22	0.278	(−0.162, 0.626)	0.210
Winter‐um	Winter‐essmax	21	0.451	(0.024, 0.739)	0.040
Spring‐rrr24	Spring‐tm	21	−0.724	(−0.880, −0.425)	0.000
Spring‐essmax	Spring‐tm	21	−0.396	(−0.707, 0.044)	0.076
Spring‐um	Spring‐tm	21	−0.772	(−0.903, −0.511)	0.000
Spring‐essmax	Spring‐rrr24	22	0.537	(0.149, 0.781)	0.010
Spring‐um	Spring‐rrr24	22	0.650	(0.314, 0.841)	0.001
Spring‐um	Spring‐essmax	22	0.327	(−0.110, 0.658)	0.137
Summer‐rrr24	Summer‐tm	22	−0.205	(−0.577, 0.237)	0.361
Summer‐um	Summer‐tm	22	−0.924	(−0.968, −0.822)	0.000
NDVI	Summer‐tm	22	0.593	(0.229, 0.812)	0.004
Summer‐um	Summer‐rrr24	22	0.312	(−0.127, 0.648)	0.158
NDVI	Summer‐rrr24	22	−0.010	(−0.430, 0.413)	0.965
NDVI	Summer‐um	22	−0.536	(−0.781, −0.148)	0.010

Abbreviations: essmax, Maximum snow depth (mm); rrr24, Daily rainfall (mm); tm, Temperature (°C); um, Humidity (%).

Subsequently, all selected variables were initially included in the model separately. Following model selection procedures and significance testing (*p* < 0.005), only the most influential variables, summer NDVI, maximum snow depth in spring (Spring‐essmax), and average winter temperature (Winter‐tm) were retained. These key predictors were then incorporated into the final model to assess their individual and combined effects on the population dynamics of the Caspian red deer.

### 3.4 Climate Variables and Red Deer Population Dynamics

All selected weather variables from different seasons were initially included separately in the model to examine their potential influence on red deer population dynamics. Following model selection based on statistical significance (*p* < 0.005), only the most influential predictors summer—NDVI, spring maximum snow depth, and winter average temperature were—retained, while non‐significant variables were excluded. These key variables were then incorporated into the final model to evaluate their effects on red deer population trends. The results revealed that winter temperature and vegetation productivity (NDVI) had a significant positive effect on population size, whereas higher spring snow depth negatively affected red deer populations.

### 3.5 Model Summary

The model accounted for 72.33% of the variance (*R* ^2^ = 72.33%), with an adjusted *R* ^2^ of 67.45%, indicating a stable and robust model with minimal bias due to the number of predictors. The model identified higher winter temperature, vegetation productivity (NDVI), and spring snow depth as key ecological drivers influencing population fluctuations. The model's performance was further supported by information criteria values, with an Akaike Information Criterion corrected for small sample sizes (AICc) of 156.42 and a Bayesian Information Criterion (BIC) of 156.99. The closeness of these values reflects an optimal balance between model complexity and goodness‐of‐fit, confirming the suitability of the selected model structure. Nonetheless, the remaining unexplained variance likely reflects additional ecological factors not included in the model.

1. NDVI: NDVI was found to have a significant positive effect on the population size of Caspian red deer (*p* = 0.003, *T* = 3.74).
2. Winter Temperature: Winter temperature showed a significant positive effect on the population size of Caspian red deer (*p* = 0.013, *T* = 2.68).
3. Spring Maximum Snow Depth: Spring maximum snow depth was found to have a significant negative effect on the population size of Caspian red deer (*p* = 0.008, *T* = −2.68).

Fitted Equations:

The marginal fitted equation and conditional fitted equation for predicting the red deer population are as follows:

Marginal Fitted Equation:

$$\begin{array}{cc} \text{Population}= & -181.6+7.73\;\text{Winter}-\mathrm{tm}–0.0719\;\text{Spring}\\ & -\text{essmax}+877\;\text{NDVI} \end{array}$$

These equations summarize the relationship between the red deer population and the climatic variables included in the analysis. They are useful for predicting how changes in temperature, vegetation productivity, and snow depth might influence red deer populations in the future.

To examine temporal trends in these variables, we used data from three meteorological stations: Siah Bisheh, Nowshahr, and Kojur. We performed linear regression analyses using year as the independent variable and climatic parameters as dependent variables. The results showed that the average winter temperature at the Nowshahr station has changed significantly in recent years (Table 2).

**TABLE 2:** Linear regression results for trends in key climatic parameters across the three studied meteorological stations over the study period.

Weather station	Winter tm				Spring essmax			
	*p*	*R* ^2^_adj_	*F*	df	*p*	*R* ^2^_adj_	*F*	df
Siah Bisheh	0.19	4%	1.85	20	0.43	0%	0.65	21
Nowshahr	0.001	28.4%	12.5	29	—	—	—	—
Kojur	0.086	13.8%	3.4	15	0.083	13.2%	3.4	16

**FIGURE 6:**
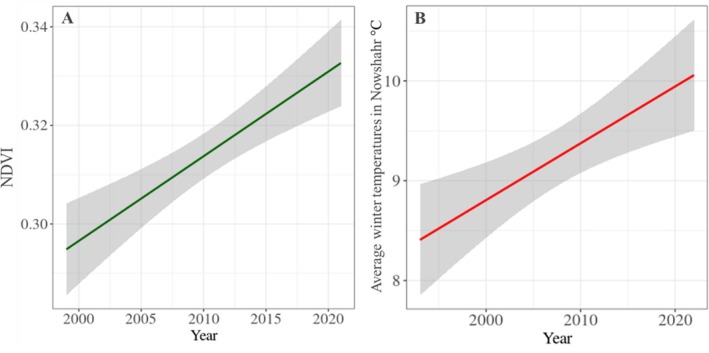
The regression results show an increase in winter temperatures in Nowshahr.

In addition to the winter temperature at Nowshahr, which showed a significant upward trend over the past two decades (*p* = 0.001, *R* ^2^ = 28.4%; *F* = 12.5; df = 29), NDVI representing vegetative productivity as a non‐climatic factor affecting the Caspian red deer also exhibited a significant increase. Linear regression analysis indicated a steady rise in NDVI values (*P* < 0.001, *R* ^2^ = 53.9%; *F* = 25.52; df = 21), corresponding to an average annual increase of approximately 0.6% (Figure 6).

**FIGURE 7:**
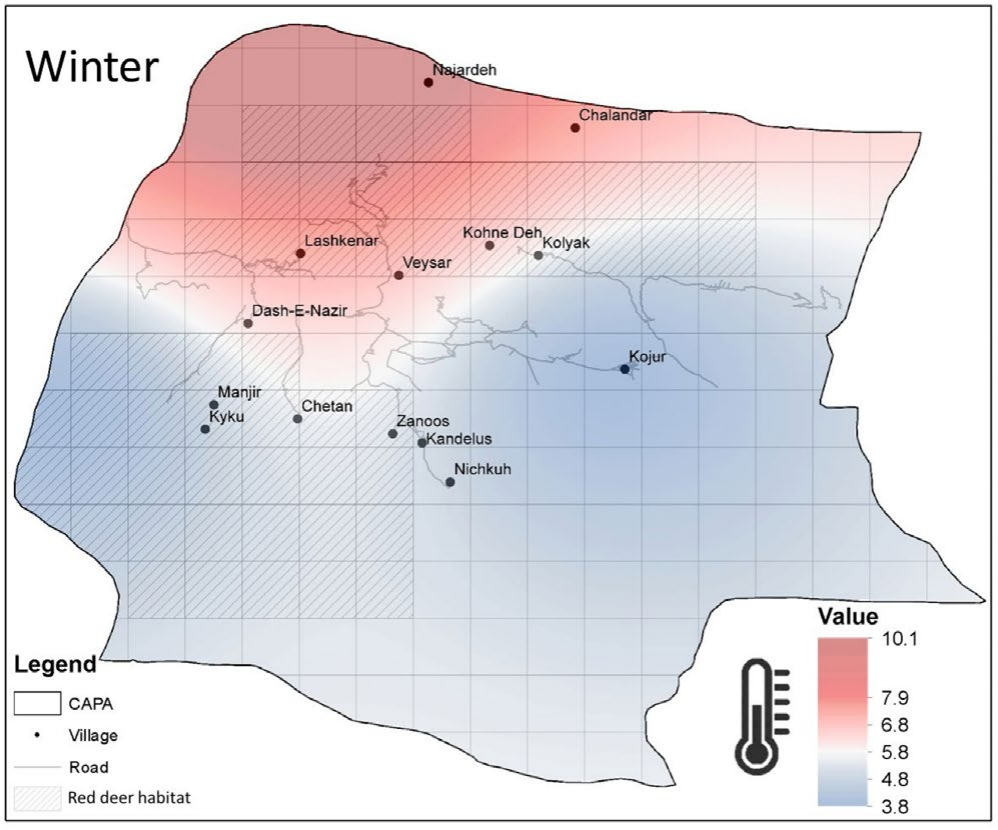
Seasonal temperature (°C) maps for winter in the Central Alborz Protected Area.

Our findings showed that from 2018 to 2022, winter temperatures varied significantly across the CAPA. The northern low‐elevation forest habitats, with milder winter temperatures averaging around 10.1°C, exhibited higher temperatures compared to the southern highlands, where average temperatures dropped to 3.8°C during the same period. These results highlight the critical role of low‐elevation forest habitats in the southern part of the region as areas with relatively higher temperatures, providing thermally favorable conditions during the harsh winter months (Figure 7).

## 4 Discussion

Our findings show that the Caspian red deer population in the Golestanak core area has grown annually by ~2% over the past two decades, with notable trends in the male and female populations, which increased by ~2.1% and ~2.2%, respectively. In contrast, the calf population exhibited a substantial growth of ~3%, contributing to an increase in the calf‐to‐female ratio. This ratio rose significantly from ~0.20 to 0.25 calves per female in the first 5 years to ~0.35–0.36 in the last 5 years, while the male‐to‐female ratio remained stable. Furthermore, our analysis revealed that winter temperatures and NDVI had a positive effect on population size, while spring snowfall negatively affected it. Despite limited seasonal changes in weather patterns at Siah Bisheh, regional variations in altitude within the Central Alborz Protected Area (CAPA) were evident at the three meteorological stations. Winter temperatures notably increased at the Nowshahr station. The milder northern regions, characterized by warmer winters and less spring snowfall, provide critical winter shelter, supporting red deer survival and seasonal migration. These findings highlight the importance of low‐elevation northern forest habitats as vital refuges for the species.

Our findings reveal a positive correlation between increased higher winter temperatures and deer population size. Temperature variability across CAPA's altitudinal gradient has resulted in a notable increase in spring and winter temperatures, particularly in lower‐altitude habitats that function as key wintering grounds for deer from early autumn to late spring. This pattern is consistent with the findings of Bonnet et al. (2019), Froy et al. (2019), Pérez‐Barbería et al. (2020), Jiang et al. (2020), and Lovari et al. (2020), which indicate that higher seasonal temperatures enhance food resource availability for herbivores, supporting increased survival rates, particularly among calves. Moreover, research by Lovari et al. (2020), Apollonio and Chirichella (2023), Salmanpour et al. (2023), and LaSharr et al. (2023) further corroborates this, demonstrating that temperature significantly influences plant phenology, thereby impacting various aspects of deer nutrition ecology. These findings highlight the critical role of seasonal temperature increases in supporting population resilience by enhancing access to resources during key developmental stages. However, it is important to note that while warmer spring and winter temperatures can generally benefit deer populations, this relationship may be disrupted when rainfall is insufficient. For example, studies on moose have shown that warm springs combined with low rainfall can negatively affect food availability and, consequently, population dynamics (Holmes et al. 2021). Additionally, contrasting findings in other red deer populations indicate that warmer summers may negatively affect body condition and recruitment, primarily due to heat stress and parasitism (Moyes et al. 2011; Stopher et al. 2014; Corlatti et al. 2018; Handeland et al. 2019). Similarly, other cervid species have exhibited similar patterns, with rainfall limitations undermining the positive effects of warmer temperatures (Felton et al. 2024). In this study, we did not observe a significant effect of rainfall on deer demographics, which may indicate that precipitation has not yet been strongly influenced by climate change in this region.

The consistent sex ratio of approximately 5–6 females per male observed throughout the study period suggests that the Golestanak core zone provides a suitable and stable habitat for Caspian red deer (Kruuk et al. 1999; Mysterud et al. 2000). Despite the documented impacts of climate change in the broader region, this stable sex ratio reflects a resilient population that has experienced notable growth over the past two decades. Situated within a climate change hotspot, Iran faces increasing threats to habitats and native species, particularly large mammals (Jowkar et al. 2016; Rahimi et al. 2019; Yousefi et al. 2019; Ebrahimi et al. 2021; Yusefi et al. 2021; Zahed et al. 2022; Naqinezhad et al. 2022). However, our findings show a significant increase in calf survival rates within CAPA, with the calf‐to‐female ratio rising from ~1:5.9 in 1995 to ~1:2.6 in 2022, indicating a marked improvement in reproductive success. This positive trend is likely driven by a combination of favorable weather conditions and improved forage availability during critical periods for calf survival (Kerby and Post 2013; Bonnet et al. 2019; Froy et al. 2019; Renaud et al. 2019; Paoli et al. 2020; Pérez‐Barbería et al. 2020; Jiang et al. 2020; Lovari et al. 2020; Millán et al. 2021).

Our findings suggest a complex interaction between temperature fluctuations and the population structure of Caspian red deer. The observed effects of warmer winters and springs with less snowfall on red deer population dynamics are consistent with broader ecological patterns in northern herbivore species, where favorable weather conditions enhance food availability and reduce energy demands for thermoregulation (Loison and Langvatn 1998; Fieberg et al. 2008; Monteith et al. 2011; Weiskopf et al. 2019; Kautz et al. 2020; Anderwald et al. 2021). Warmer winters reduce energy expenditure, allowing more resources to be allocated to growth and reproduction, while warmer springs promote earlier vegetation growth, leading to improved nutrition during calving season. This, in turn, enhances reproductive success and calf survival rates (Loison et al. 1999; Pérez‐Barbería et al. 2020; Jiang et al. 2020). Conversely, the negative effects of spring snowfall on the population are consistent with studies showing that deeper snow hampers foraging efficiency and increases energy expenditure for locomotion, leading to reduced survival (Pépin et al. 2009; Simard et al. 2014; Seto et al. 2015). This highlights the importance of snowfall during transitional seasons in shaping red deer reproductive timing and population trends in temperate regions (Loe et al. 2021; Laurent et al. 2021).

The observed annual population increase of approximately 2.1% in the red deer population within the Golestanak core zone a—primary finding of this study stands—in contrast to the ~2% annual decline reported over the past four decades across northern Iran (Kiabi et al. 2004; Shokri et al. 2021). We hypothesize that this population growth, while partly attributable to favorable climatic conditions, also reflects a combination of factors, including enhanced protection efforts and improved habitat quality following the prohibition of livestock grazing. Specifically, the establishment of the Golestanak ranger station and extensive patrols throughout CAPA to curb poaching have likely played a crucial role, as documented by Ghoddousi et al. (2016, 2019), Jones et al. (2019), Soofi et al. (2019), Yusefi et al. (2019), Shokri et al. (2021), Dobbins et al. (2020), and Milda et al. (2020), who identify poaching as the most significant threat to herbivore populations, particularly the Caspian red deer in northern Iran. Additionally, CAPA's unique topography, characterized by high elevations, diverse vegetation, and rugged, rocky terrain in the southern regions, offers a natural refuge that further supports red deer populations by reducing accessibility for poachers. The prohibition of livestock grazing within Golestanak has likely contributed to habitat restoration, reducing competition for resources, as suggested by Soofi et al. (2018), who found that livestock presence negatively impacts Caspian red deer distribution. These combined factors underscore the complex interplay between active management practices and natural landscape features in fostering a resilient red deer population within the core zone.

While climate change can drive herbivore populations toward higher elevations with more suitable habitats (Brinkman et al. 2005; Monteith et al. 2011; Lendrum et al. 2013; Van Beest et al. 2013; Lamsal et al. 2018; Salmanpour et al. 2022; Eom et al. 2023), its impact may be relatively positive for red deer in CAPA due to favorable habitat conditions and effective conservation strategies. The success of CAPA underscores the importance of proactive management in reversing population declines, particularly in regions facing significant anthropogenic pressures.

The dramatic increase of an endangered large herbivore species sensitive to climate change in the past two decades, such as the Caspian red deer in CAPA, further highlights the importance of upland grassland conservation. This growth is linked to enhanced ranger presence, effective conservation strategies, and favorable climatic conditions that support food availability and calf survival. Increasing the area of the Golestanak core zone, with a particular emphasis on the high pastures of Zanoos, is essential for providing critical refuge for deer and ensuring access to vital resources during seasonal migrations. Moreover, the identification and restoration of migration corridors connecting wintering and summering grounds are crucial for facilitating seasonal movements and ensuring long‐term population resilience. Addressing anthropogenic barriers, such as the Kojur road and nearby villages, is vital for maintaining habitat connectivity and mitigating the impacts of climate change. Therefore, comprehensive conservation strategies that integrate local community engagement, habitat protection, and proactive management are imperative for the sustained survival of the Caspian red deer and the ecological integrity of their habitats.

## 5 Conclusion

Our study highlights the significant role of environmental factors in shaping the population dynamics of Caspian red deer in the Central Alborz Protected Area (CAPA). Higher winter temperatures and increased NDVI were found to positively influence the red deer population, while spring snowfall had a detrimental effect, reducing population growth. The findings emphasize the critical role of protecting both high‐elevation habitats during summer and low‐elevation forest habitats during winter. The northern low‐elevation forested areas of CAPA are particularly vital in winter, providing essential shelter and supporting the deer's survival during harsh conditions. In contrast, the southern highlands, with their favorable temperatures, are critical for red deer in summer. Protecting and restoring corridors between these seasonal habitats is essential to ensure the persistence of the Caspian red deer population. These measures will help mitigate the impacts of climate change and preserve the ecological integrity of the region.

## Author Contributions

**Farid Salmanpour:** conceptualization (lead), data curation (equal), formal analysis (equal), investigation (equal), methodology (equal), project administration (lead), resources (equal), software (lead), supervision (lead), validation (lead), visualization (lead), writing – original draft (lead), writing – review and editing (lead). **Zahra Shakoori:** data curation (equal), formal analysis (equal), investigation (equal), methodology (equal), resources (equal), software (equal), writing – review and editing (equal). **Mahan Salmanpour:** data curation (equal), writing – review and editing (equal). **Mehdi Kia:** data curation (equal), writing – review and editing (equal). **Rahman Eshaghi:** data curation (equal), writing – review and editing (equal). **Abolfazl Rahbarizadeh:** data curation (equal), writing – review and editing (equal). **Faraham Ahmadzadeh:** supervision (equal), validation (equal), visualization (equal), writing – review and editing (equal).

## Ethics Statement

The authors have nothing to report.

## Consent

The authors have nothing to report.

## Conflicts of Interest

The authors declare no conflicts of interest.

## Data Availability

Datasets analyzed during the current study are available on Figshare as https://figshare.com/s/dd87622d5232b928d416.
